# Are Individuals Luck Egalitarians? – An Experiment on the Influence of Brute and Option Luck on Social Preferences

**DOI:** 10.3389/fpsyg.2017.00460

**Published:** 2017-03-29

**Authors:** Gustav Tinghög, David Andersson, Daniel Västfjäll

**Affiliations:** ^1^JEDILab, Division of Economics, Department of Management and Engineering, Linköping UniversityLinköping, Sweden; ^2^Department of Medical and Health Sciences, The National Center for Priority Setting in Health Care, Linköping UniversityLinköping, Sweden; ^3^Decision Research, EugeneOR, USA

**Keywords:** fairness, luck egalitarianism, brute luck, option luck, strategy method dictator game, laboratory experiment

## Abstract

According to luck egalitarianism, inequalities should be deemed fair as long as they follow from individuals’ deliberate and fully informed choices (i.e., option luck) while inequalities should be deemed unfair if they follow from choices over which the individual has no control (i.e., brute luck). This study investigates if individuals’ fairness preferences correspond with the luck egalitarian fairness position. More specifically, in a laboratory experiment we test how individuals choose to redistribute gains and losses that stem from option luck compared to brute luck. A two-stage experimental design with real incentives was employed. We show that individuals (*n* = 226) change their action associated with re-allocation depending on the underlying conception of luck. Subjects in the brute luck treatment equalized outcomes to larger extent (*p* = 0.0069). Thus, subjects redistributed a larger amount to unlucky losers and a smaller amount to lucky winners compared to equivalent choices made in the option luck treatment. The effect is less pronounced when conducting the experiment with third-party dictators, indicating that there is some self-serving bias at play. We conclude that people have fairness preference not just for outcomes, but also for how those outcomes are reached. Our findings are potentially important for understanding the role citizens assign individual responsibility for life outcomes, i.e., health and wealth.

## Introduction

How to deal fairly with the burdens and benefits that follow from individuals’ fortune and misfortune has long been a prominent topic in philosophy (Nagel, 1979; Williams, 1981; Levy, 2011). This discussion is also closely linked with policy issues such as; when should individuals be held financially responsible for their own ill health? to what extent should society level out the inequalities in financial wealth? This paper seeks to investigate how different types of luck influence social preferences in a behavioral experiment.

### Brute and Option Luck

It is evident that aspects of luck significantly affect outcomes in many spheres of life. The genes that we are equipped with at birth are highly influential in determining the extent to which we will be able to live a long and healthy life. The social environment that we are born into heavily influences our future wealth, etc. Such outcomes can be ascribed to *brute luck*, i.e., how risks fall out that are *not* in the sense deliberate choices. However, not all of life’s outcomes are simply due to such unforeseeable brute luck. In fact, most choices that we make involve deliberate risk-taking, e.g., buying a lottery ticket, biking in traffic, and smoking cigarettes. The outcomes associated with such choices are a matter of how deliberate choices that involve risk-taking turn out – whether or not our *option luck* is good or bad. The extent to which society should seek to eliminate inequalities that are due to these two different types of luck is a recurrent topic in philosophy and public policy. However, to our knowledge, no experiment has investigated the extent to which individuals’ fairness preferences about inequalities change due to different types of luck.

The distinction between brute luck and option luck was first introduced by Ronald Dworkin (Dworkin, 1981), who stated that:

Option luck is a matter of how deliberate and calculated gambles turn out – whether someone gains or loses through accepting an isolated risk he or she should have anticipated and might have declined. Brute luck is a matter of how risks fall out that are not in the sense deliberate gambles. (Dworkin, 1981, p. 293). If I buy a stock on the exchange that rises, then my option luck is good. If I am hit by a falling meteorite whose course could not have been predicted, then my bad luck is brute (even though I could have moved just before it struck if I had had any reason to know where it would strike) (Dworkin, 1981, p. 73).

The concept of brute luck and option luck later became closely associated with what is sometimes labeled as luck egalitarianism (Arneson, 1989; Cohen, 1989). The standard formulation of this doctrine is that a person should not be worse off than anyone else, in respect to some metric or currency of goods, as a result of brute luck. Thus, inequalities for which individuals have had no possibility to influence through their own choices should be deemed unfair and therefore equalized, while inequalities that follow from individuals’ deliberate and fully informed choices should be deemed fair. The traditional distinction between inequity and inequality is intimately linked to the notion of brute and option luck. There could be equity in an unequal distribution if inequalities arise from option luck, but when an unequal distribution arises from brute luck inequality and inequity coincides.

### Previous Experiments

Behavioral experiments have consistently established that individuals care not only about their own material payoff, but they also care about other-regarding aspects (Forsythe et al., 1994; Andreoni et al., 2003; Camerer, 2003; Engel, 2011). The dictator game^1^ is a workhorse for studying fairness preferences in experiments since it involves no strategic concerns related to behavior. The first dictator game with real stakes was conducted by Forsythe et al. (1994) who found that around 60% of participants shared a positive amount of money, with the mean transfer at roughly 20% of the endowment. These findings of the standard dictator game have since been replicated numerous times and appear to be fairly robust also across cultures (Henrich et al., 2001). The growth of this empirical literature^2^ has fueled theoretical advances in economic models that aim to explain observed non-selfish behavior. The inequity-aversion models of Fehr and Schmidt (1999) and Bolton and Ockenfels (2000) have been established as the paramount theoretical models to explain so-called social preferences in dictator games (Fehr and Schmidt, 1999; Bolton and Ockenfels, 2000). These models focus on individuals’ relative position to explain non-selfish behavior. Another category of fairness models assigns a major behavioral role to reciprocity, i.e., intentions (e.g., Rabin, 1993; Dufwenberg and Kirchsteiger, 2004; Falk and Fischbacher, 2006). However, both the inequity-aversion and reciprocity models have maintained a consequentialist perspective, focusing on distributive fairness concerns while largely neglecting the fact that individuals also have preferences regarding how these outcomes are reached – procedural fairness concerns^3^.

Most experimental studies that have investigated individuals’ concerns for procedural fairness have been designed to examine how effort and ability influence distributive choices. This is typically done in a two-person bargaining game by assigning a proposer based on some form of unrelated ability or effort measure (e.g., a hash-mark game or a word-search task). The assigned responder then gets to allocate an initial entitlement between himself/herself and a respondent who may accept or decline this offer. The general conclusion from these studies is that most people consider distributive inequalities as fair as long as they stem from differences in effort or ability (Hoffman and Spitzer, 1985; Burrows and Loomes, 1994; Konow, 2000; Frohlich et al., 2004; Cappelen et al., 2007, 2010, 2014). Similarly, Rodriguez-Lara and Moreno-Garrido (2012) show that individuals in a dictator game typically endorse a biased ideal of fairness and employ justice principles in a self-serving way to maximize their own payoff.

Although the influence of effort and ability on fairness preferences, have received considerable attention, less attention has been devoted to different aspects of luck and how it influences fairness preferences in behavioral experiment. At the macro level, a strong correlation between how much a country spends on social programs and its citizens’ beliefs about whether luck or effort determines wealth has been established (Fong, 2001; Alesina and Angeletos, 2005; Isaksson and Lindskog, 2009). The two studies that come closets to the behavioral experiment we present here are the studies by Möllerström et al. (2015) and Cappelen et al. (2013).

Cappelen et al. (2013) employed a two-stage experiment to study fairness views about risk-taking. The first phase involved participants making a sequence of choices between risky and safe alternatives in a gambling situation. In the second phase, participants were paired and the earnings of each pair were pooled. Participants were informed about the choices and outcome of the risk-taking phase for both parties and were asked to distribute total earnings. Cappelen et al. (2013) concluded that most participants in their sample endorsed ex post redistribution between lucky and unlucky risk takers, but not between risk-takers and participants who avoided risk and chose a safe alternative. They label this fairness position *choice egalitarianism*. Although choice egalitarianism is closely related to luck egalitarianism, the design employed by Cappelen et al. (2013) did not allow for the essential comparisons between different types of luck and how this influences individuals’ preferences for redistribution. To do this, it is necessary to incorporate aspects of voluntary and involuntary risk-taking.

Möllerström et al. (2015) also employed a two-stage experiment in which third-party spectators redistributed resources between agents who could partly insure themselves against unfavorable outcomes. Each spectator decided whether to leave earnings unchanged or equalize them in 11 potential scenarios involving the same agents. Thus, there was a joint precence of uncontrollable and controllable events that spectators had to consider when making redistributive decisions. Möllerström et al. (2015) found that spectators were more willing to redistribute money when bad brute luck was causing the outcome. However, they also found that spectators condition compensation for bad brute luck on agents’ irrelevant choices about option luck exposure. Thus, they conclude that people are more accurately described as “choice compensators” than luck egalitarians. For example, choice compensators would hold smokers more responsible for poor health than non-smokers regardless of whether the disease is caused by smoking or not.

The main objective of this study is to investigate the extent to which individuals’ preferences for redistribution correspond with the luck egalitarian fairness position in situations which separately involve brute and option luck. The primary hypothesis, which we set out to test, is:

Hypothesis: Individuals equalize inequalities resulting from brute luck to a greater extent compared to inequalities resulting from option luck.

In addition to the stated hypothesis, which follows directly from luck egalitarianism and primarily focus on different types of bad luck, we also set out to investigate if different types of good luck influence individuals’ preferences for redistribution. This matter is less frequently discussed. However, if individuals choose to redistribute less money to individuals who suffer bad option luck it would be analogous if individuals were also more willing to redistribute money to individuals who enjoy good option luck, given that these are also outcomes from deliberate choices. Also, in capitalist market economies, taking economic risks is an essential part of the role of entrepreneurs. Hence, we also want to investigate if individuals reward successful, deliberate risk-taking.

Finally, we set out to investigate if fairness views related to luck is dependent on individuals being involved in the actual experiment. Previous studies have proposed that differences in stakeholder-view make little difference for individuals when making fairness judgment (Konow, 2000, 2009). However empirical evidence on this matter remains scarce. By comparing the fairness behavior between third-party dictators and dictators actively taking part in the decision-making phase, we aim to examine the extent to which fairness views of stakeholders deviate from impartial spectators with regards to brute and option luck.

The remainder of the paper is organized as follows. The Section “Materials and Methods” describes the basic design of the experiments and the data collection procedure. The Section “Result” presents the results from our experiment. The Section “Discussion” discusses policy implications and potential caveats associated with our experimental design.

## Materials and Methods

Two separate experiments to investigate how different types of luck influence social preferences were conducted. These experiments were identical except that Experiment 1 relied on fairness judgments made by participants with personal stake related to the outcome of the luck-related task, while Experiment 2 included third-party participants to make the equivalent fairness judgment. Both experiments involved self-selected students as participants. Although worries about the external validity of such a subject pool could be raised, studies by for example Exadaktylos et al. (2013) have shown that self-selected students are an appropriate subject pool for the study of social behavior.

### Experiment 1

Participants were recruited among students at the Department of Management and Engineering at Linköping University. In total, 126 subjects (49% Females; Mean age 22) participated in 16 experimental sessions that lasted approximately 20 min. No one participated more than once, and individuals with prior knowledge about the experiment were excluded from the experiment. Prior to the experiment, subjects were randomly assigned to either option luck or brute luck treatment. We ran equal numbers of brute and option luck experiment sessions on a given day. Every second session was either option or brute luck treatment. Subjects were allowed to specify if there were any particular times when they were unable to participate. No information concerning payment for participating in the experiment was given beforehand. The experiment was single blind, i.e., the participant could not associate any decisions with particular subjects, but one experimenter oversaw the decisions in order to arrange payments. Payments were made in cash in a marked envelope at the end of the experiment.

Before each experimental session, we randomly paired two individuals and assigned them to a role of either dictator or recipient in the dictator game. Hence, we employed a random dictator rule where each person had an equal chance of dictating the result, and strategic considerations were eliminated. All instructions were presented in written form. Subjects were asked not to talk to other participants during the experiment, but were encouraged to raise questions to the experiment leader if anything was unclear. For complete instructions see Supplementary Material.

The experiment was divided into three phases; the initial *treatment phase* where subjects were presented with instructions associated with either option or brute luck, the *allocation phase* where subjects made allocation decisions before the actual outcome of the experiment was disclosed, and the final *outcome phase* where a coin toss was used to separate lucky winners and unlucky losers.

At the outset of the experiment, subjects in the option luck treatment were given a two-option choice for how they would be compensated. If subjects chose the safe option, they received 50 SEK for participating in the experiment. If they chose the risky option a coin toss (executed by the experiment leader) would settle if the subject would receive 150 SEK or 0 SEK for participating in the experiment. Hence, the expected value of the risky option was 75 SEK. Given that the expected payoff was 50% higher if choosing the risky option, it would arguably be perceived as the “right” choice. At the outset of the experiment, subjects in the brute luck treatment were told that their compensation would be settled through a coin toss (150 SEK or 0 SEK) at the end of the experiment. Hence, the expected value associated with participating was 75 SEK.

In the strategy phase, subjects were informed that they were anonymously and randomly paired with another participant, and that one participant in each pair would randomly be awarded an additional 100 SEK. The subject was asked to specify how he/she would distribute these additional 100 SEK between himself/herself and the anonymous partner by means of the strategy method, i.e., subjects were asked to specify their actions for every possible scenario (presented in the same order) in the experiment prior to knowing the outcome of the coin toss. This allowed us to collect data on a subject’s complete strategy. In the brute luck treatment, the two potential scenarios were that the subject was paired with a winner (150 SEK) or a loser (0 SEK). Subjects in the option luck treatment faced the same potential scenarios, but in addition they were also asked to specify how they would distribute 100 SEK in a scenario where they were paired with someone who had chosen the safe alternative (50 SEK) in the initial treatment phase. Given that this was a one-shot experiment, subjects did not have to consider how their distribution could potentially affect future outcomes.

When everyone had specified their dictator strategies, these were collected and a coin toss was used to separate lucky winners from unlucky losers. The experimenter publicly executed the coin toss. Once this was done, the subjects were asked to fill out an unrelated questionnaire while one of the experimenters went to a separate room to arrange envelopes with correct payment, based on the strategies revealed.

### Experiment 2

The procedure and methods used in Experiment 2 followed the same structure as in Experiment 1. The only difference in design between Experiments 1 and 2 concerned the inclusion of third-party dictators to make the allocation decision. Thus, the allocation phase in experiment 2 was conducted with impartial subjects who did not themselves participate in the actual task involving different types of luck. In total, 200^4^ subjects (37% Females; Mean age 22) participated in four sessions that lasted approximately 15 min.

Subjects were randomly assigned to one of four possible roles; Brute Luck Receiver; Option Luck Receiver; Brute Luck Dictator, Option Luck Dictator. Participants with respective roles conducted the experiment in separate rooms. Dictators were randomly paired with a receiver from their respective treatment. Dictators received 50 SEK as a show-up fee and did the same unrelated fill-in task as all other participants in the experiment. Finally, dictators were asked to split 100 SEK between themselves and their randomly assigned partner according to the same strategy method used in Experiment 1.

### Analysis

To test our hypothesis with regard to the difference in redistribution rates between the brute luck and the option luck treatments, an unpaired two-sample *t*-test was conducted. To test differences with regard to the difference in redistribution rates between gamblers and non-gamblers in the option luck treatment a paired two-sample *t*-test was conducted. The results from the *t*-tests were confirmed via a non-parametric bootstrap analysis.

### Ethics Statement

We consulted the ethical review board for East Sweden to determine whether a formal approval of the committee was required. It was concluded that a formal assessment by the Ethics Committee was not necessary because the participants were given fulldisclosure of the procedure (i.e., there was no deceit), participants received a payment proportionate to the task at hand, the experimental procedure was noninvasive and the results were analyzed anonymously. Furthermore, the participants in all experiments were recruited online through our JEDILab subject pool and voluntarily subscribed for participation in the described experiments. They were informed participation was voluntary and anonymous.

## Results

**FIGURE 1:**
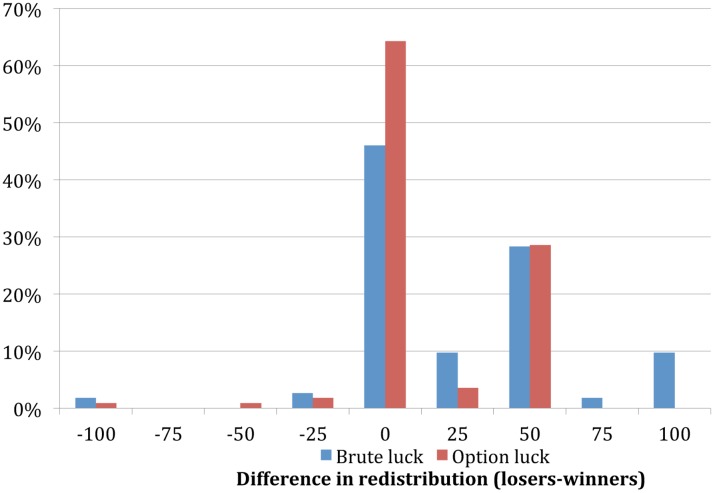
**Distribution of responses for the difference in redistribution between losers and winners in each experimental treatment**.

**Table 1** presents descriptive results from Experiments 1 and 2. The pooled results clearly show that procedural justice concerns related to option and brute luck influence an individual’s preferences for redistribution. On average subjects in the brute luck treatment gave more to unlucky losers than to lucky winners (on average 24.2 SEK more). Subjects in the option luck treatment gave only 12.5 SEK more to unlucky losers compared to lucky winners. This difference between experimental treatments in how subjects redistributed money was statistically significant [*t*(223) = 2.73, *p* = 0.0069]. As shown in **Figure 1** this difference was mainly due to the higher share (64.3% vs. 46.0%) of subjects in the option luck treatment who made no difference in amount redistributed for losers and winners.

**Table 1:** Redistribution in the dictator game for option and brute luck.

	Brute luck (mean SEK Sent by dictators)	Option luck (mean SEK sent by dictators)	Difference (Brute luck-Option luck)	*P*-value*t*-test	*P*-value Mann–Whitney
*Experiment 1*					
Unlucky losers (0 SEK)	47.0	38.1	8.9	0.0733	0.2229
Lucky winners (150 SEK)	21.6	27.3	–5.6	0.2533	0.1449
Difference (losers-winners)	25.3	10.8	14.5	0.0191	0.0349
*Experiment 2 with third-party dictators*					
Unlucky losers (0 SEK)	36.3	33.6	2.8	0.5983	0.8681
Lucky winners (150 SEK)	13.7	19.1	–5.4	0.2810	0.1071
Difference (losers-winners)	22.7	14.5	8.2	0.1718	0.1170
*Pooled*					
Unlucky losers (0 SEK)	42.4	36.1	6.30	0.0839	0.3061
Lucky winners (150 SEK)	18.2	23.6	–5.4	0.1268	0.0378
Difference (losers-winners)	24.2	12.5	11.70	0.0069	0.0089


As shown in **Table 1**, the average redistribution rates that subjects chose for participants who lost the coin toss (unlucky losers) was higher in the brute luck treatment (42%) compared to the option luck treatment (36%), suggesting stronger social preferences for individuals who suffer bad brute luck compared to bad option luck [*t*(223) = 1.73, *p* = 0.0839]. This result is in line with our hypothesis that individuals redistribute a larger share of their own endowment to individuals who have suffered bad brute luck compared to bad option luck. **Table 1** also shows that participants redistribute higher amounts to winners of the coin toss (lucky winners) in the option luck treatment (24%) compared to the brute luck treatment (18%). Albeit not significant, this result suggests that outcomes due to good option luck are deemed to be fairer than outcomes due to good brute luck. When using a non-parametric test (Mann–Whitney), however, the result for lucky winners is significant (*z* = –2.08, *p* = 0.0378) while the result for unlucky losers is no longer significant at the 10%-level (*z* = 1.02, *p* = 0.3061).

Looking at the results from Experiment 2 alone we see that the difference between treatments are no longer significant [*t*(97) = 1.38, *p* = 0.1718]. However, a further interaction analysis showed that the difference in the effect of treatment did not significantly differ between the experiments (see Table A1–A3 in Supplementary Materials). All effect signs in Experiment 2 go in the same direction as in Experiment 1, indicating that stakeholders and spectators in general act on the same fairness views. In addition we also see that third-party dictators in general give significantly less to both losers [*t*(223) = 2.10, *p* = 0.0368] and winners [*t*(223) = 2.27, *p* = 0.0242] compared to dictators in Experiment 1. Thus, third-party dictators show weaker altruistic preferences.

**Table 2** shows that subjects in Experiment 1 chose to redistribute significantly less to risk averse non-gambling individuals compared to both unlucky losers and lucky winners. Consequently, non-gamblers were punished by participants as a consequence of their cautious behavior^5^. These findings are in line with the findings from Cappelen et al. (2013) who found that people equalized earnings significantly less in distributive situations in which risk takers were paired with participants choosing a safe option. In Experiment 2 we see the same effect when comparing risk averse individuals with unlucky losers. However, for lucky winners the effect surprisingly goes in the opposite direction (albeit not significant) compared to Experiment 1, i.e., third-party dictators give more to non-gamblers than lucky winners.

**Table 2:** Redistribution to risk averse subjects (i.e., non-gamblers) compared to risk takers in the option luck treatment.

	Value	Diff	*P*-value
*Experiment 1*			
Risk averse (non-gamblers) – Unlucky losers	18.4–38.1	–19.7	<0.0001
Risk averse (non-gamblers) – Lucky winners	18.4–27.3	–8.9	0.0245
*Experiment 2*			
Risk averse (non-gamblers) – Unlucky losers	24.6–33.6	–9.0	0.0059
Risk averse (non-gamblers) – Lucky winners	24.6–19.1	5.6	0.1009
*Experiments 1 and 2 (Pooled)*		
Risk averse (non-gamblers) – Unlucky losers	21.2–36.0	–14.9	<0.0001
Risk averse (non-gamblers) – Lucky winners	21.2–23.6	–2.4	0.3644


## Discussion

Which inequalities among individuals should be considered unjust and therefore equalized? The doctrine of luck egalitarianism proposes that when individuals are worse off than others because of bad brute luck, they should have a claim to compensation, whereas if their disadvantage can be traced back to specific choices they made deliberately, then the inequality appears justified. To many solidarity reaches its limit when neediness is self-inflicted. However, our results show that this is true also for risky behavior. Individuals do not only judge the same outcome distribution differently, but change their action associated with re-allocation depending on the underlying conception of luck. This study provides better understanding for how individuals evaluate social outcomes where luck has been at play, but where factors of merit/effort and entitlement have been excluded.

From a general point of view, the results from this experiment are in line with previous studies, which suggested that process-related fairness plays an important role when forming social preferences, e.g., (Frohlich et al., 1987; Bolton et al., 2005; Cappelen et al., 2007). In this respect our results also cast serious doubt on the consequentialistic practice inherent in standard economic theory that focuses solely on utility related to outcomes while neglecting the underlying process.

From a more specific point of view, the findings from this experiment demonstrate that individuals change their behavior depending on the type of luck underlying inequalities. Moreover, we see a behavioral pattern that redistribution is higher when inequalities are due to brute luck. This suggests that the general luck egalitarian fairness view is not just a philosophic endeavor, but also a theory that is in accordance with the fairness judgments made by a non-negligible fraction of the population in our sample.

Our main hypothesis focused on inequalities due to bad luck, suggesting that individuals have stronger social preferences toward individuals who have suffered bad brute luck compared to bad option luck. The results show higher redistribution among individuals randomly assigned to the brute luck treatment, suggesting stronger other-regarding preferences toward individuals who suffer bad brute luck compared to bad option luck in our study sample.

Our analysis relates to the study by Cappelen et al. (2013) who investigated the impact of risk-taking on social preferences. However, Cappelen et al. (2013) investigated social preferences in a context of ex ante equality in opportunities, but ex post inequalities in earnings. By imposing an exogenous shift between option and brute luck, the design of this study allows us to take the opposite approach and investigate social preferences in a context of ex ante inequality in opportunities and equality in ex post earnings. The findings by Cappelen et al. (2013) show that inequalities between lucky and unlucky risk takers are deemed more acceptable than inequalities between risk takers and people choosing the safe alternative – a finding that is in line with what we find in our option luck treatment.

Although observed patterns with regards to other-regarding preferences are similar in Experiments 1 and 2, it should be noted that fairness preferences are less strongly in line with luck egalitarianism in Experiment 2 where we employ third-party dictators. However, a further interaction analysis showed that the difference in the effect of treatment did not significantly differ between the experiments. Interestingly we also find that third-party dictators show less altruistic preferences when redistributing money in the dictator game compared to dictators who make identical decisions from a stakeholder view. This finding is robust across experimental treatments and goes against previous studies by Konow (2000, 2009) and Cappelen et al. (2013) who have argued that differences in stakeholder-view make little difference for individuals when making fairness judgments.

As stated at the outset of this paper, the issues we address are in essence normative. Still, empirical insight concerning how individuals’ social preferences for inequalities are formed and influenced is key for understanding the formation and sustainability of any welfare system. If policies that seek to level inequalities that most individuals think are fair is implemented, it could potentially erode the feeling of solidarity necessary for a well functioning welfare system. Hence, the findings from this study are relevant for understanding a wide range of public choices where aspects of luck are in play, e.g., public bailouts in situations of financial crisis and financial redistribution for different types of income.

The other side of the influence of luck is, of course, responsibility. To what extent should individuals be held responsible for favorable/unfavorable outcomes? For luck egalitarians the response is that any outcome not derived from brute luck should be attributed to individual responsibility. However, in real life it is not easy to make a clear distinction between brute and option luck. Much of the political discourse on funding for health care has centered on the role of individual responsibility in healthcare financing (Buyx, 2008; Tinghög et al., 2010). Is a cancer patient with a history of heavy smoking less entitled to public insurance compared to a non-smoking cancer patient? The results from our experiment by no means settle complicated questions like these. But the experiment provides empirical background, which could feed the normative debate. Our study might, however, shed some light on why there seems to be an increasing trend toward assigning individual responsibility an explicit role in public policy. Obviously, luck egalitarian thinking underlies the taxation of risky activities that could potentially lead to outcomes costly for society, e.g., use of tobacco, alcohol, and unhealthy food. Moreover, insurance policies in principle a structured arrangement where the lucky compensate the unlucky.

## Author Contributions

All authors listed, have made substantial, direct and intellectual contribution to the work, and approved it for publication.

## Conflict of Interest Statement

The authors declare that the research was conducted in the absence of any commercial or financial relationships that could be construed as a potential conflict of interest.

## Footnotes

1 The game typically involves two players; a dictator and a receiver. The anonymous dictator makes an initial choice on how to allocate (split) some endowment and the anonymous receiver passively receives the remainder of the endowment. The strategy for a person motivated purely by material self-interest would be to keep the entire endowment and leave the receiver with nothing.

2 At the time of writing this article, the empirical literature on dictator games comprises more than 129 published articles; see Engel (2011) for a meta-study.

3 We here define procedural fairness broadly; as concerns how the distribution of individuals’ prospects or opportunities should be allowed to causally influence material outcomes.

4 Hundred subjects participated as receivers only in the specially designed version of the dictator game.

5 It should be noted that the risky option had a higher expected value and that this is likely to have influenced the result. For future research, it would thus be interesting to explore similar situations where expected value are identical for risky and safe options.

## References

[B1] Alesina A. Angeletos G. M. (2005). Fairness and redistribution. *Am. Econ. Rev.* 95 960–980. 10.1257/0002828054825655

[B2] Andreoni J. Harbaugh W. Vesterlund L. (2003). The carrot or the stick: rewards, punishments, and cooperation. *Am. Econ. Rev.* 93 893–902. 10.1257/000282803322157142

[B3] Arneson R. J. (1989). Equality and equal opportunity for welfare. *Philos. Stud.* 56 77–93. 10.1007/BF00646210

[B4] Bolton G. E. Brandts J. Ockenfels A. (2005). Fair procedures: evidence from games involving lotteries^∗^. *Econ. J.* 115 1054–1076. 10.1111/j.1468-0297.2005.01032.x

[B5] Bolton G. E. Ockenfels A. (2000). ERC: a theory of equity, reciprocity, and competition. *Am. Econ. Rev.* 90 166–193. 10.1257/aer.90.1.166

[B6] Burrows P. Loomes G. (1994). The impact of fairness on bargaining behavior. *Empir. Econ.* 19 201–221. 10.1007/BF01175872

[B7] Buyx A. M. (2008). Personal responsibility for health as a rationing criterion: why we don’t like it and why maybe we should. *J. Med. Ethics* 34 871–874. 10.1136/jme.2007.024059 19043112

[B8] Camerer C. F. (2003). *Behavioral Game Theory: Experiments in Strategic Interaction*. Princeton, NJ: Princeton University Press.

[B9] Cappelen A. W. Eichele T. Hugdahl K. Specht K. Sørensen E. O. Tungodden B. (2014). Equity theory and fair inequality: a neuroeconomic study. *Proc. Natl. Acad. Sci. U.S.A.* 111 15368–15372. 10.1073/pnas.1414602111 25313056 PMC4217432

[B10] Cappelen A. W. Hole A. D. Sørensen E. Ø. Tungodden B. (2007). The pluralism of fairness ideals: an experimental approach. *Am. Econ. Rev.* 97 818–827. 10.1257/aer.97.3.818

[B11] Cappelen A. W. Konow J. Sorensen E. O. Tungodden B. (2013). Just luck: an experimental study of risk-taking and fairness. *Am. Econ. Rev.* 103 1398–1413. 10.1257/aer.103.4.1398

[B12] Cappelen A. W. Sørensen E. Ø. Tungodden B. (2010). Responsibility for what? Fairness and individual responsibility. *Eur. Econ. Rev.* 54 429–441. 10.1016/j.euroecorev.2009.08.005

[B13] Cohen G. A. (1989). On the currency of egalitarian justice. *Ethics* 99 906–944. 10.1086/293126

[B14] Dufwenberg M. Kirchsteiger G. (2004). A theory of sequential reciprocity. *Games Econ. Behav.* 47 268–298. 10.1016/j.geb.2003.06.003

[B15] Dworkin R. (1981). What is equality? Part 2: equality of resources. *Philos. Public Aff.* 10 283–345.

[B16] Engel C. (2011). Dictator games: a meta study. *Exp. Econ.* 14 583–610. 10.1007/s10683-011-9283-7

[B17] Exadaktylos F. Espın A. M. Branas-Garza P. (2013). Experimental subjects are not different. *Sci. Rep.* 3:1213 10.1038/srep01213 PMC3572448 23429162

[B18] Falk A. Fischbacher U. (2006). A theory of reciprocity. *Games Econ. Behav.* 54 293–315. 10.1016/j.geb.2005.03.001

[B19] Fehr E. Schmidt K. M. (1999). A theory of fairness, competition, and cooperation. *Q. J. Econ.* 114 817–868. 10.1098/rspb.2015.0392

[B20] Fong C. (2001). Social preferences, self-interest, and the demand for redistribution. *J. Public Econ.* 82 225–246. 10.1016/S0047-2727(00)00141-9

[B21] Forsythe R. Horowitz J. L. Savin N. E. Sefton M. (1994). Fairness in simple bargaining experiments. *Games Econ. Behav.* 6 347–369. 10.1006/game.1994.1021

[B22] Frohlich N. Oppenheimer J. Kurki A. (2004). Modeling other-regarding preferences and an experimental test. *Public Choice* 119 91–117. 10.1023/B:PUCH.0000024169.08329.eb

[B23] Frohlich N. Oppenheimer J. A. Eavey C. L. (1987). Choices of principles of distributive justice in experimental groups. *Am. J. Pol. Sci.* 31 606–636. 10.2307/2111285

[B24] Henrich J. Boyd R. Bowles S. Camerer C. Fehr E. Gintis H. (2001). In search of Homo economicus: behavioral experiments in 15 small-scale societies. *Am. Econ. Rev.* 91 73–78. 10.1257/aer.91.2.73

[B25] Hoffman E. Spitzer M. L. (1985). Entitlements, rights, and fairness – an experimental examination of subjects concept of distributive justice. *J. Legal Stud.* 14 259–297. 10.1086/467773

[B26] Isaksson A. S. Lindskog A. (2009). Preferences for redistribution–a country comparison of fairness judgements. *J. Econ. Behav. Organ.* 72 884–902. 10.1016/j.jebo.2009.08.006

[B27] Konow J. (2000). Fair shares: accountability and cognitive dissonance in allocation decisions. *Am. Econ. Rev.* 90 1072–1091. 10.1257/aer.90.4.1072

[B28] Konow J. (2009). Is fairness in the eye of the beholder? An impartial spectator analysis of justice. *Soc. Choice Welfare* 33 101–127. 10.1007/s00355-008-0348-2

[B29] Levy N. (2011). *Hard Luck: How Luck Undermines Free Will and Moral Responsibility*. Oxford: Oxford University Press 10.1093/acprof:oso/9780199601387.001.0001

[B30] Möllerström J. Reme B. A. Sørensen E. T. (2015). Luck, choice and responsibility – an experimental study of fairness views. *J. Public Econ.* 131 33–40. 10.1016/j.jpubeco.2015.08.010

[B31] Nagel T. (1979). *Mortal Questions.* London: Cambridge University Press.

[B32] Rabin M. (1993). Incorporating fairness into game-theory and economics. *Am. Econ. Rev.* 83 1281–1302.

[B33] Rodriguez-Lara I. Moreno-Garrido L. (2012). Self-interest and fairness: self-serving choices of justice principles. *Exp. Econ.* 15 158–175. 10.1007/s10683-011-9295-3

[B34] Tinghög G. Carlsson P. Lyttkens C. H. (2010). Individual responsibility for what? – a conceptual framework for exploring the suitability of private financing in a publicly funded health-care system. *Health Econ. Policy Law* 5 201–223. 10.1017/S174413310999017X 19723367

[B35] Williams B. A. O. (1981). *Moral Luck: Philosophical Papers 1973–1980.* Cambridge: Cambridge University Press 10.1017/CBO9781139165860

