# Supplementary material for: Are Individuals Luck Egalitarians? – An Experiment on the Influence of Brute and Option Luck on Social Preferences
Source: Front Psychol. 2017 Mar 29;8:460. doi: 10.3389/fpsyg.2017.00460 (PMC5372824; doi:10.3389/fpsyg.2017.00460)
Supplement: Supplementary file 1 [file Data_Sheet_1.docx]

## Supplementary Material

## Instructions

*General instructions (experiment 1 and 2, all treatments).*

The purpose of this session is to study how people make decisions in particular situations. During the session you will have the opportunity to earn money. Upon completion of the session the amount you make will be paid in cash. Payments and responses are confidential: no other participant will know this information.

All necessary information will be presented on the papers in front of you. If you have any questions, feel free to raise your hand, and a monitor will assist you. From now until the end of the session do not talk with other participants.

*Option Luck Treatment (experiment 1)*

**Stage 1**

For participating in this experiment you will receive 50 SEK. You may choose to gamble with this money. If you choose to gamble you will have a 50% chance to win 150 SEK and a 50% risk to lose your 50 SEK.

If you chose to gamble, a coin toss executed by the experiment leader will settle if you win or loose.

Do you wish to gamble?

Yes No

Before we proceed to execute the coin toss please fill out the remaining questions (Note 1: Subjects were not allowed to proceed to Stage 2 before everyone had finished Stage 1. Note 2: Subjects were not aware of stage 2 while being in stage 1)

**(NEW PAGE)**

**Stage 2**

You have been randomly paired with another person in this experiment. Neither you nor the other person will ever get to know the identity of each other. One person at random in each pair will be given an additional amount of 100 SEK, which he/she shall allocate between himself/herself and the other person

You now have to decide how you wish to allocate this money between you and the other person, in case the additional 100 SEK is given to you. The unknown/anonymous receiver has faced the same questions as you in Stage 1, i.e. whether or not he/she wishes to gamble.

We now want you to decide how much of the additional 100 SEK you wish to share in three potential scenarios: The receiver chose not to gamble and kept his/her 50 SEK in Stage 1; the receiver chose to gamble in Stage 1 and won 150 SEK; the receiver chose to gamble in Stage 1 and lost his/her 50 SEK.

If the receiver chose not to gamble in Stage 1, your decision for this scenario will be carried out. If the receiver chose to gamble in Stage 1 and won 150 SEK, your decision for this scenario will be carried out. If the receiver chose to gamble in Stage 1 and lost his/her 50 SEK, your decision for this scenario will be carried out.

Note that there is a 50% possibility that you will become the receiver in this experiment; your randomly assigned partner will then decide how to allocate the additional 100 SEK between himself/herself and you (in the same way as described above).

How do you allocate 100 SEK between you and the randomly drawn person who chose not to gamble with the 50 SEK in Stage 1 (i.e. received 50 SEK in Stage 1)?

Me__________SEK

The other person__________SEK

How do you allocate 100 SEK between you and the randomly drawn person who chose to gamble with the 50 SEK and lost (i.e. received 0 SEK in Stage 1)?

Me__________SEK

The other person__________SEK

How do you allocate 100 SEK between you and the randomly drawn person who chose to gamble with the 50 SEK and won (i.e. received 150 SEK in Stage 1)?

Me__________SEK

The other person__________SEK

*Brute luck treatment (experiment 1)*

**Stage 1**

For participating in this experiment 50% of the participants will receive 150 SEK while the other 50% will receive 0 SEK.

Who will receive 150 SEK and 0 SEK will be decided through a coin toss, which the experiment leader will execute at the end of the experiment.

Before we proceed to execute the coin toss please fill out the questions in front of you.

**(NEW PAGE)**

**Stage 2**

You have been randomly paired with another person in this experiment. Neither you nor the other person will ever get to know the identity of each other. One person at random in each pair will be given an additional amount of 100 SEK, which he/she shall allocate between himself/herself and the other person.

You now have to decide how you wish to allocate the money between you and the other person, in case the additional 100 SEK is given to you.

The unknown/anonymous receiver will, just as you, be paid according to the outcome of the coin toss. We now want to you to decide how much of the additional 100 SEK you wish to share in two potential scenarios: The receiver won 150 SEK in Stage 1; the receiver lost and got 0 SEK in Stage 1.

If the receiver won 150 SEK in Stage 1, your decision for this scenario will be carried out. If the receiver lost and got 0 SEK in Stage 1, your decision for this scenario will be carried out.

Note that there is a 50% possibility that you will become the receiver in this experiment; your randomly assigned partner will then decide how to allocate the additional 100 SEK between himself/herself and you (in the same way as described above)

How do you allocate 100 SEK between you and the randomly drawn person who lost the coin toss in Stage 1 (i.e. received 0 SEK in Stage 1)?

Me__________SEK

The other person__________SEK

How do you allocate 100 SEK between you and the randomly drawn person who won the coin toss in Stage 1 (i.e. received 150 SEK in Stage 1)?

Me__________SEK

The other person__________SEK

*Option luck treatment (experiment 2)*

**Stage 1**

For participating in this experiment you will receive 50 SEK.

**(NEW PAGE)**

**Stage 2**

You have been randomly paired with another person in this experiment. Neither you nor the other person will ever get to know the identity of each other. You have randomly been given an additional amount of 100 SEK, which you shall allocate between yourself and the other person.

You now have to decide how you wish to allocate the additional 100 SEK between you and the other person. The unknown/anonymous receiver has faced the same questions as you in Stage 1.

Just as you, the other person has received 50 SEK for participating in the experiment. Unlike you, the other person could choose to forgo this 50 SEK and instead participate in a gamble. If he/she choose to gamble payment for the doing the initial task is decided through a coin toss, where there is 50% chance to get 150 SEK and a 50 % risk to get 0 SEK

We now want you to decide how much of the additional 100 SEK you wish to share in three potential scenarios: The receiver chose not to gamble and kept his/her 50 SEK; the receiver chose to gamble and won 150 SEK; the receiver chose to gamble and lost his/her 50 SEK.

If the receiver chose not to gamble, your decision for this scenario will be carried out. If the receiver chose to gamble and won 150 SEK, your decision for this scenario will be carried out. If the receiver chose to gamble and lost his/her 50 SEK, your decision for this scenario will be carried out.

How do you allocate 100 SEK between you and the randomly drawn person who chose not to gamble with the 50 SEK (i.e. received 50 SEK)?

Me__________SEK

The other person__________SEK

How do you allocate 100 SEK between you and the randomly drawn person who chose to gamble with the 50 SEK and lost (i.e. received 0 SEK)?

Me__________SEK

The other person__________SEK

How do you allocate 100 SEK between you and the randomly drawn person who chose to gamble with the 50 SEK and won (i.e. received 150 SEK)?

Me__________SEK

The other person__________SEK

*Brute luck treatment (experiment 2)*

**Stage 1**

For participating in this experiment you will receive 50 SEK.

**(NEW PAGE)**

**Stage 2**

You have been randomly paired with another person in this experiment. Neither you nor the other person will ever get to know the identity of each other. You have randomly been given an additional amount of 100 SEK, which you shall allocate between yourself and the other person.

You now have to decide how you wish to allocate the additional 100 SEK between you and the other person. The unknown/anonymous receiver has faced the same questions as you in Stage 1.

Unlike you, the other person’s payment for participating in the experiment is decided through a mandatory gamble. His/her payment for the doing the initial task is decided through a coin toss, where there is 50% chance to get 150 SEK and a 50 % risk to get 0 SEK

We now want to you to decide how much of the additional 100 SEK you wish to share in two potential scenarios: The receiver won 150 SEK; the receiver lost and got 0 SEK.

If the receiver won 150 SEK , your decision for this scenario will be carried out. If the receiver lost and got 0 SEK your decision for this scenario will be carried out.

How do you allocate 100 SEK between you and the randomly drawn person who lost the coin toss (i.e. received 0 SEK)?

Me__________SEK

The other person__________SEK

How do you allocate 100 SEK between you and the randomly drawn person who won the coin toss in (i.e. received 150 SEK)?

Me__________SEK

The other person__________SEK

## Additional regression analysis

*Table A1. OLS regression and interaction analysis of redistribution to Unlucky losers.*

|  | Model 1 | | Model 2 | |
| --- | --- | --- | --- | --- |
|  | Estimate | p | Estimate | p |
| Female (ref=Male) | 4.13 | 0.2635 | 4.03 | 0.2756 |
| Age | 0.61 | 0.4395 | 0.67 | 0.3960 |
| Brute luck (ref=Option luck) | 5.63 | 0.1240 | 8.30 | 0.0883 |
| Experiment 2 (ref=Experiment 1) | -6.80 | 0.0688 | -3.68 | 0.4849 |
| Brute*Experiment 2 |  |  | -6.13 | 0.4040 |

*Table A2. OLS regression and interaction analysis of redistribution to Lucky winners.*

|  | Model 1 | | Model 2 | |
| --- | --- | --- | --- | --- |
|  | Estimate | p | Estimate | p |
| Female (ref=Male) | -0.35 | 0.9227 | -0.35 | 0.9233 |
| Age | -0.29 | 0.7106 | -0.29 | 0.7115 |
| Brute luck (ref=Option luck) | -5.13 | 0.1495 | -5.18 | 0.2747 |
| Experiment 2 (ref=Experiment 1) | -8.15 | 0.0252 | -8.21 | 0.1113 |
| Brute*Experiment 2 |  |  | 0.10 | 0.9887 |

*Table A3. OLS regression and interaction analysis of difference (losers-winners) in redistribution.*

|  | Model 1 | | Model 2 | |
| --- | --- | --- | --- | --- |
|  | Estimate | p | Estimate | p |
| Female (ref=Male) | 4.48 | 0.3082 | 4.38 | 0.3198 |
| Age | 0.90 | 0.3408 | 0.96 | 0.3100 |
| Brute luck (ref=Option luck) | 10.77 | 0.0139 | 13.48 | 0.0205 |
| Experiment 2 (ref=Experiment 1) | 1.36 | 0.7595 | 4.52 | 0.4714 |
| Brute*Experiment 2 |  |  | -6.23 | 0.4760 |
